# From Pulse to Phenotype: Sleep Apnea Endotyping for Polygraphy via Oximeter-Derived Autonomic Arousal

**DOI:** 10.1016/j.chest.2025.12.005

**Published:** 2025-12-18

**Authors:** Christian Strassberger, Jan Hedner, Scott A. Sands, Ding Zou, Ludger Grote

**Affiliations:** a Center for Sleep and Vigilance Disorders, Institute of Medicine, Sahlgrenska Academy, University of Gothenburg, Gothenburg, Sweden; b Center for Sleep Medicine, Department of Respiratory Medicine, Sahlgrenska University Hospital, Gothenburg, Sweden; c Division of Sleep and Circadian Disorders, Brigham and Women's Hospital and Harvard Medical School, Boston, MA

**Keywords:** arousal threshold, autonomic arousal, collapsibility, endotypes, phenotypes, loop gain, muscle compensation

## Abstract

**Background:**

Understanding the underlying cause of OSA in the individual patient, referred to as pathophysiologic endotyping, is essential for personalized care. The current classification of these traits from routine sleep recordings relies on manually scored arousals from sleep. Automating this process could widen the applicability of endotyping.

**Research Question:**

Can analyzing autonomic variability, derived from finger oximeter photoplethysmography (PPG), accurately classify sleep apnea pathophysiologic endotypes with results comparable with the established EEG-based method?

**Study Design and Methods:**

Eighty-seven patients referred for suspected OSA underwent ambulatory polysomnography. Pulse wave amplitude, pulse rate, and pulse propagation time were derived from the PPG pulse waveform. A logistic mixed-effect model was developed to predict the presence of EEG-based arousals after respiratory events using these PPG-derived parameters, together with the associated event type and blood oxygen saturation. The automatically predicted PPG-based arousals then were incorporated into an established model (Phenotyping Using Polysomnography) to determine OSA endotypic traits from the airflow and PPG-based signals. The agreement between endotypes with PPG-based and EEG-based arousals was assessed using intraclass correlation coefficient (ICC).

**Results:**

PPG-derive pulse wave responses to respiratory events were more pronounced in the presence of arousal (*P* < .001 for all). The model using PPG metrics to predict cortical arousal demonstrated moderate performance (sensitivity, 0.71; specificity, 0.59). Endotypic traits derived by PPG-derived arousals showed strong agreement compared with the EEG-derived reference (ICC for loop gain at 1 cycle/min, 0.95 [95% CI, [0.88-0.98]; ICC for ventilation at active muscle activity, 0.96 [95% CI, 0.81-0.99]; ICC for ventilation at passive muscle activity, 0.99 [95% CI, 0.99-0.99]; ICC for ventilation at minimum muscle activity, 0.99 [0.99-0.99]; ICC for arousal threshold, 0.85 [95% CI, 0.48-0.96]; ICC for muscle compensation, 0.80 [95% CI, 0.60-0.91]).

**Interpretation:**

Our results show that using pulse wave features instead of manually scored EEG-based arousals in respiratory modelling allows for accurately determining OSA endotypes. This approach might enable physiologic endotyping in non-EEG-based sleep studies, expanding the accessibility of personalized OSA management.

**Take-Home Points**

**Research Question:** Can analyzing autonomic variability through oximeter-derived pulse wave features accurately classify sleep apnea pathophysiologic endotypes with results comparable with those of established EEG-based methods?

**Results:** Pulse wave features demonstrate a significant response to sleep arousals in patients with OSA, and autonomic arousals derived from this analysis facilitate automated OSA endotyping in strong agreement with the traditional polysomnography-based Phenotyping Using Polysomnography model.

**Interpretation:** This research indicates that using autonomic arousals from pulse wave analysis offers a standardized and fully automated method for OSA endotyping, applicable even with simpler home sleep apnea testing devices.

OSA is a highly prevalent disorder with diverse manifestations and significant cardiovascular and metabolic consequences. Although traditionally attributed to upper airway obstruction, knowledge of disease mechanisms and their pathophysiologic features in OSA is increasing. Specifically, 4 main mechanisms have been identified to describe its pathophysiologic characteristics^1^: passive anatomic features, upper airway muscle activity, arousal threshold, and loop gain. Quantifying these mechanisms or traits to characterize patient subgroups better (ie, pathophysiologic endotyping) has been described to facilitate precision medicine for patients with OSA. Indeed, increasing evidence suggests that pathophysiologic endotyping gives insights into treatment responses^2^^,^^3^ or the prediction of treatment success.^4^^,^^5^ Notably, gold standard approaches to measure these traits involve invasive measurement of ventilatory drive or complex study protocols to manipulate drive, which are not feasible for routine clinical practice.^6^, ^7^, ^8^

Several years ago, a method was developed to enable pathophysiologic endotyping to be applied in routine polysomnography (Phenotyping Using Polysomnography [PUP]^9^, ^10^, ^11^). Using a simplified model of the respiratory control system, the time course of ventilatory drive was estimated from the observed ventilation during sleep. Importantly, the model uses EEG-based arousals as a covariate so that the resulting loop gain trait captures the chemical drive, rather than the ventilatory response to arousals. Furthermore, the arousal threshold is derived as the average ventilatory drive before each manually scored arousal. Metrics describing collapsibility and muscle compensation are based on breaths identified during sleep in the absence of arousals. With the increasing demand for simpler, cost-efficient, multinight assessments for diagnostic purposes in clinical routine, EEG often is unavailable.^12^^,^^13^ Thus, these simplified assessments without EEG recordings are unsuitable for endotypic characterizations using established models like PUP. Therefore, routine assessments of OSA endotypes outside clinical research trials are limited.

Autonomic responses to changes in cortical activity are well established.^14^^,^^15^ Several methods were proposed to classify arousals from sleep using changes in the finger blood flow, heart rate responses, and skin conductance.^16^^,^^17^ Although these approaches may have limited accuracy when compared with manual scorings of cortical arousals from EEG, autonomic features have been linked to OSA severity and consequences.^18^, ^19^, ^20^ This suggests that the autonomic response may be linked to the ventilatory response to arousals in OSA, positioning autonomic features as a potential tool to evaluate endotypic characteristics. Therefore, we hypothesized that incorporating autonomic variability as a proxy of arousal classification, defined by oximeter-derived pulse wave features, can determine pathophysiologic endotypes accurately compared with the established PUP model using EEG recordings.

## Study Design and Methods

### Study Cohort

The data for this evaluation were taken from a cross-sectional study designed to classify the pathophysiologic features of OSA through detailed recordings of vital signs, blood samples, and sleep. Ninety-seven participants were referred for suspected sleep apnea evaluation at the Sahlgrenska University Hospital Sleep Center, accepted study participation, and provided written and oral consent. They underwent a single standardized ambulatory polysomnographic sleep assessment, a medical examination, and sleep-related questionnaires. The study was performed according to the Declaration of Helsinki for clinical trials, and the protocol was approved by the local ethics committee (Identifier: 2019-05096).

### Sleep Study

The single-night ambulatory polysomnography (Nox A1; Nox Medical) included a complete montage of EEG, electrooculography, chin and left and right anterior tibialis electromyography, and ECG, together with recordings of finger pulse oximetry, respiratory effort, and nasal-oral pressure. Patients with missing or poor-quality signals in nasal-oral pressure, finger pulse oximetry, or EEG (defined as > 75% artifacts during sleep duration) were excluded from further analyses. The sensors were attached carefully in the sleep center by trained specialists, and the patient slept at home.

All recordings were manually scored by 1 certified sleep technician using the standards set by the American Academy of Sleep Medicine.^21^ Hypopnea classification used a ≥ 3% desaturation criterion, cortical arousal, or both. The frequency of apnea and hypopnea events per hour was captured and expressed as the apnea-hypopnea index. At least 4% desaturation events were used to determine the oxygen desaturation index. For each respiratory event, additional metrics such as the duration and the association with arousals and desaturations were classified. The nadir in the oxygen saturation after the event was determined, and the difference from the baseline before the event was recorded as the associated blood oxygen saturation (SpO_2_) reduction.

### Photoplethysmography Analysis

**Figure 1:**
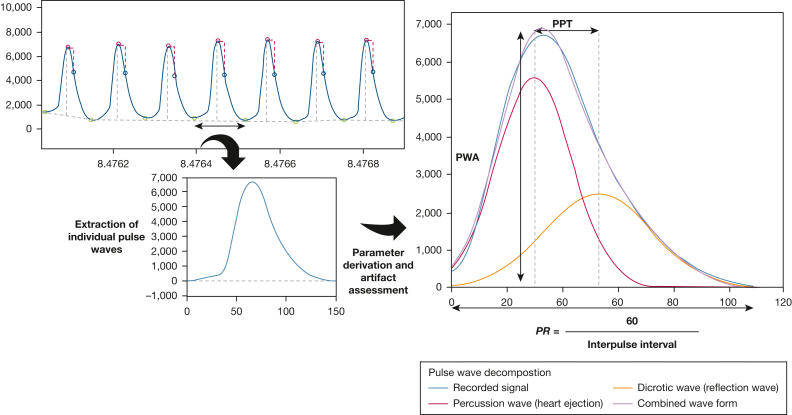
Diagrams showing an overview of the procedure to derive pulse wave characteristics. Pulse waves are detected, extracted, and approximated individually using a curve-fitting algorithm. The resulting decomposition of the pulse wave into percussion wave and dicrotic wave is used to derive the characteristics of the pulse wave. PPT = pulse propagation time; PR = pulse rate; PWA = pulse wave amplitude.

The pulse waveform signal was derived from photoplethysmography (PPG), using the Nonin WristOx2 3150 oximeter (Nonin Medical B.V., as part of the Nox A1 system). The evaluation was performed using an in-house signal processing routine developed for this project (e-Appendix 1). In brief, a low-pass filter was applied to assist the initial detection of individual pulse beats. Next, a decomposition of the pulse wave was performed, as applied in other contexts previously.^22^ For this purpose, a model-based curve-fitting approach was applied to the original signal. Subsequently, pulse rate (PR), pulse wave amplitude (PWA), and pulse propagation time (PPT) were derived on a beat-to-beat basis, visualized in Figure 1 and e-Figure 1. Individual beat-to-beat pulse wave features were combined into a continuous signal trace, with linear interpolation for sequences of up to 5 invalid pulse waves (e-Fig 2).

### Derivation of Autonomic Responses

Respiratory event-related responses in pulse wave features (PR, PWA, and PPT) were derived in accordance with previous definitions of sleep apnea-related changes in PPG-derived metrics^23^, ^24^ (e-Appendix 1). In brief, this approach is based on a patient-specific search window surrounding the respiratory event (e-Fig 3). The search window was defined based on the ensemble average of the pulse wave signal centered at the end of the respiratory events. For each event, the autonomic response was defined as the difference between the post-event maximum and the event minimum for PR (ie, PR surge), or the post-event minimum and event maximum for PWA and PPT (ie, PWA and PPT drop). The event-specific reduction in PWA corresponds to the previously defined vasoconstriction drop, VCD_EV_.^24^

Data from individual events related to pulse wave responses in PR, PWA, and PPT were pooled for further analyses. Additionally, based on previous evaluations of event-related variation in autonomic responses, associated SpO_2_ reductions and type of event (apnea or hypopnea) were recorded for each event.^25^ These factors then were included in a prediction model to classify the presence or absence of event-related arousals. The model-predicted arousals were termed autonomic arousals.

### Derivation of Endotypic Traits

Endotypic traits were derived according to the methodology described in detail earlier using an automated analysis tool (PUP version 08/2024).^9^, ^10^, ^11^ In brief, the nasal-oral pressure signal was used as input, together with manually scored apnea, hypopnea, and arousal events. Based on the ventilatory responses to airway obstructions, ventilatory drive was estimated. Nine distinct endotypic traits were computed and used in the final evaluation. These traits reflect the ventilatory control system (ie, loop gain at 1 cycle/min and loop gain at natural frequency, arousal threshold, the ventilatory response to arousals, and time delay), upper airway anatomic features (ie, ventilation at minimum muscle activity [V_min_], ventilation at active muscle activity [V_active_], ventilation at passive muscle activity [V_passive_]), and muscle compensation (muscle compensation = V_active_-V_passive_). This established methodology, utilizing manual EEG-based arousal scoring, is in the following referred to as PUP_EEG_.

The approach was modified to substitute EEG-based arousals with PPG-based autonomic responses: Before the evaluation of endotypic traits, the probability of arousal presence was computed for each respiratory event based on the statistical prediction model (see Statistical Analysis) and was classified as autonomic arousals. The duration of autonomic arousal was chosen to cover 4 breaths after the respiratory event. This rationale is based on previous data on arousal contribution to ventilatory drive.^26^ This scoring of autonomic arousals then was used to derive endotypic traits instead of the manual scoring of arousals using EEG. This modified approach is in the following referred to as PUP_PPG_.

### Statistical Analysis

The statistical analyses and visualizations were performed using R version 4.3.3 software (R Foundation for Statistical Computing). Pooled respiratory event data were evaluated to document the autonomic responses to events in the presence vs absence of arousals. Descriptive data are presented as mean (SD) or median (interquartile range), where appropriate, based on data distribution assessed via normality testing (skewness, Anderson-Darling test, visual inspection). The typical time course of changes in autonomic variables was visualized using an ensemble average, performed separately for each event type (apnea and hypopnea, with vs without arousal), with respiratory events dynamically normalized in time.

To predict the presence or absence of arousals after respiratory events, a mixed-effects logistic regression model was developed using a designated training data set consisting of 80% of patients (n = 70). This model incorporated patient-specific random intercepts to account for interindividual variability and potential within-patient correlations in autonomic responses, together with the predictors PR, PWA, PPT, SpO_2_ reduction, and type of event. Final predictions were based on the model’s fixed effects, representing the estimated response for an average member of the population. To determine a prediction cut off, the Youden index was calculated, which optimizes the balance of sensitivity and specificity derived from the receiver operating characteristic curve.^27^ Recognizing the imbalance in the prevalence of arousals after respiratory events (approximately 80%), and assuming the cost of missing an arousal (false-negative result) to be higher than a false-positive result, a weighted Youden’s index was selected. This approach involved weighting sensitivity 1.25 times stronger, prioritizing higher sensitivity in the model’s predictions.

Endotypic traits derived using the modified PUP_PPG_ method, using autonomic arousals, were compared against the benchmark established PUP_EEG_ measurements in a designated test set (n = 17). Paired *t* tests, intraclass correlation coefficients (ICCs), and Bland-Altman statistics were used. Additional sensitivity analyses are provided in e-Appendix 1, including associations with confounders, assessment of multicollinearity, evaluation of the prediction cut off, and variations by sleep stage and position. Furthermore, the quantification of the arousal threshold was evaluated in the context of clinical predictors.^28^

## Results

### Demographics

Data from 10 patients were excluded from this analysis because of missing or low-quality signals (loss of flow or PPG signal, signal artifacts, or nonidentifiable pulses). Characteristics of the analysis cohort (n = 87) are presented in Table 1. Patients (74% male; mean (SD) age, 54 (12) years; and mean (SD) BMI, 27.7 (3.6) kg/m^2^) demonstrated moderate to severe sleep apnea (mean (SD) apnea-hypopnea index, 33 (23) events/h) and demonstrated mean (SD) systolic BP of 133 (12) mm Hg and mean (SD) diastolic BP of 79 (9) mm Hg.

**Table 1:** Baseline Characteristics of the Primary Analysis Cohort

Variable	Data
No. of patients	87
Age, y	54 (12)
Female sex	23 (26.4%)
Current smoking	35 (40.2%)
Diabetes	6 (6.9%)
Hypertension	21 (24.1%)
BMI, kg/m^2^	27.7 (3.6)
BP, mm Hg	
Systolic	133 (12.0)
Diastolic	79 (9.1)
AHI, events/h	32.6 (22.8)
ODI, events/h	10.3 (4.2-21.1)

Data are presented as No. (%), mean (SD), or median (interquartile range). AHI = apnea-hypopnea index; ODI = oxygen-desaturation index.

### Autonomic Responses

**Figure 2:**
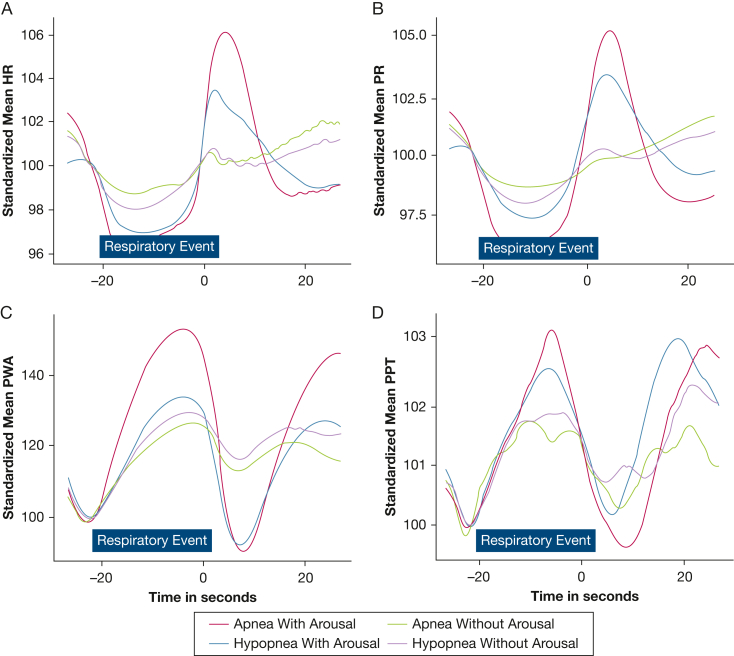
Graphs showing responses in autonomic signals, divided by event type and the presence of arousals according to standardized mean HR (A), standardized mean PR (B), standardized mean PWA (C), and standardized mean PPT (D). Data shown are pooled ensemble averages of all respiratory events, with respiratory events stretched to match a standardized duration of 20 seconds. Time = 0 represents event termination. Although HR and PR show steep increases after the termination of events, PWA and PPT show reductions in these signals. The presence of arousals enhances the response in all tested parameters. HR = heart rate; PPT = pulse propagation time; PR = pulse rate; PWA = pulse wave amplitude.

An overview of the pooled respiratory event data and the associated responses in PR, PWA, PPT, and SpO_2_ is given in Table 2. A total of 18,275 respiratory events comprising 7,450 apneas (central, n = 336; mixed, n = 670; obstructive, n = 6,444) and 10,825 hypopneas were recorded, of which 79% (33% apnea and 46% hypopnea) terminated with manually scored EEG-arousals. As hypothesized, events accompanied by manually scored arousals were associated with a marked change in autonomic responses. The type of respiratory event showed a less pronounced impact on the observed autonomic responses. Apneas without arousals were followed by a 42% (hypopneas, 41%) reduction in PWA, whereas the presence of arousals led to a 55% (hypopneas, 50%) reduction. A visualization of the signal traces in the studied autonomic signals (PR, PWA, and PPT) with or without associated EEG-arousals is shown in Figure 2, using pooled ensemble averages with normalized event duration.

**Table 2:** Overview of Cardiovascular Responses for All Respiratory Events, Pooled From 87 Recordings

Variable	Type of Event				Presence of Arousal			
	Apnea			Hypopnea	Apnea		Hypopnea	
	Central	Mixed	Obstructive		With	Without	With	Without
No. (%)	336 (2%)	670 (4%)	6,444 (35%)	10,825 (59%)	6,086 (33%)	1,364 (7%)	8,382 (46%)	2,443 (13%)
Event duration, s	16.3 (13.3-19.9)	29.5 (23.8-39.1)	21.0 (14.6-31.0)	19.4 (14.9-26.5)	23.8 (16.3-33.5)	15.0 (12.0-20.4)	19.6 (15.0-26.9)	18.9 (14.6-25.4)
SpO_2_ change, %	4 (2-5)	7 (5-9)	5 (3-8)	2 (1-4)	5 (3-8)	3 (2-5)	2 (1-4)	3 (2-4)
HR change, %	15.6 (9.3-23.2)	21.2 (10.9-33.4)	17.1 (9.9-27.3)	13.9 (8.6-22.6)	19.2 (11.4-29.9)	10.8 (7.0-16.6)	15.6 (9.7-25.2)	9.9 (6.5-14.9)
PR change, %	9.8 (5.5-16.8)	16.8 (8.2-27.9)	12.7 (6.8-21.4)	9.7 (5.6-16.8)	14.5 (7.9-23.7)	7.4 (4.7-12.3)	10.8 (6.2-18.8)	6.9 (4.1-11.4)
PWA change, %	–44.1 (19.0)	–57.7 (15.2)	–52.0 (17.0)	–47.7 (18.5)	–54.5 (15.9)	–41.8 (18.3)	–49.8 (17.9)	–40.5 (18.6)
PPT change, %	–22.5 (11.9)	–23.8 (10.9)	–20.0 (11.1)	–18.9 (11.0)	–21.1 (11.4)	–17.9 (10.1)	–19.4 (11.3)	–17.0 (9.9)

Data are presented as No. (%), mean (SD), or median. HR = ECG-derived heart rate; PPT = pulse propagation time; PR = photoplethysmography-derived pulse rate, PWA = pulse wave amplitude; SpO_2_ = blood oxygen saturation.

### Association of Arousals With Autonomic Variables

**Figure 3:**
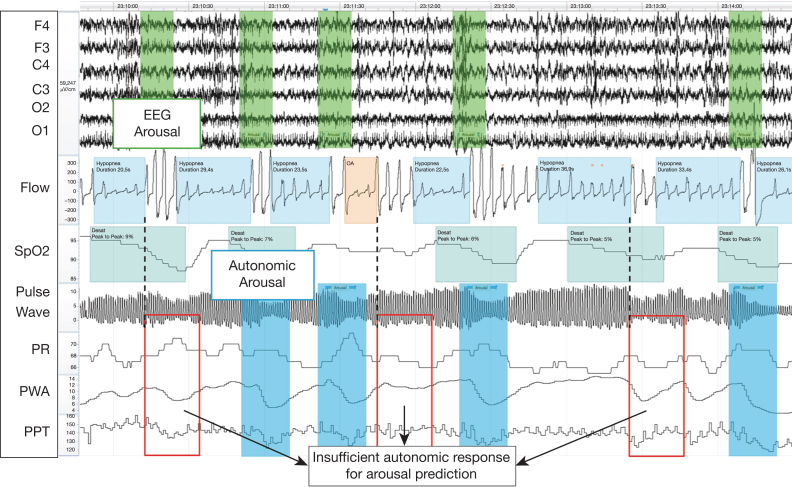
Case example of an oximeter-derived autonomic arousal prediction vs manual EEG scoring. This excerpt from a single patient displays key signals (EEG, respiratory flow, SpO_2_, photoplethysmography waveform, and derived PR, PWA, and PPT) and highlights manually scored EEG arousals (green shaded areas), predicted autonomic arousals (blue shaded areas), and negative predictions (red squares). In this segment, 7 respiratory events are determined, of which 6 are classified correctly (4 of 5 respiratory events with and 2 respiratory events without subsequent arousals). PPT = pulse propagation time; PR = pulse rate; PWA = pulse wave amplitude; Spo_2_ = blood oxygen saturation.

All autonomic and hypoxic event responses were associated significantly with manually scored EEG arousals, adjusted for confounders (e-Table 1). The final model to predict autonomic arousals as part of the PUP model pipeline used only coefficients for the PPG-derived autonomic and hypoxic responses, together with the event type. Sensitivity analyses confirmed low to moderate collinearity between predictors, with no indication of variance inflation (e-Tables 2, 3). During the model training, the Youden index was found at 0.806, with a sensitivity of 0.63 and a specificity of 0.67. The final weighted prediction cut off was chosen at 0.745, with sensitivity of 0.77 and specificity of 0.51 (e-Table 4, e-Fig 4). On the test data set, the model reached a sensitivity and specificity of 0.71 and 0.59, respectively. True and false predictions of arousals for training and test sets are shown in Table 3. In the test set analysis, 65% of respiratory events were predicted to lead to an arousal. An additional visualization of predicted probabilities, separated by event, is provided in e-Figure 5. A case example illustrating the oximeter-derived arousal prediction against manual EEG scoring, including instances of correct detections and discrepancies, is presented in Figure 3.

**Table 3:** True or False Predictions of the Presence of Arousals After Apnea and Hypopnea Events in the Training (n = 70 Patients) and Test (n = 17 Patients) Data

Variable	Autonomic: No Arousal	Autonomic: Arousal
Training data (n = 70)		
EEG: No arousal	1,611	1,522
EEG: Arousal	2,633	8,734
Test data (n = 17)		
EEG: No arousal	347	242
EEG: Arousal	730	1,775

In training and test data, 71% and 65%, respectively, of events are classified as terminated by arousal, with sensitivity and specificity of 0.77 and 0.51, respectively, and of 0.71 and 059, respectively.

### Comparison of PUP_EEG_ and PUP_PPG_

**Figure 4:**
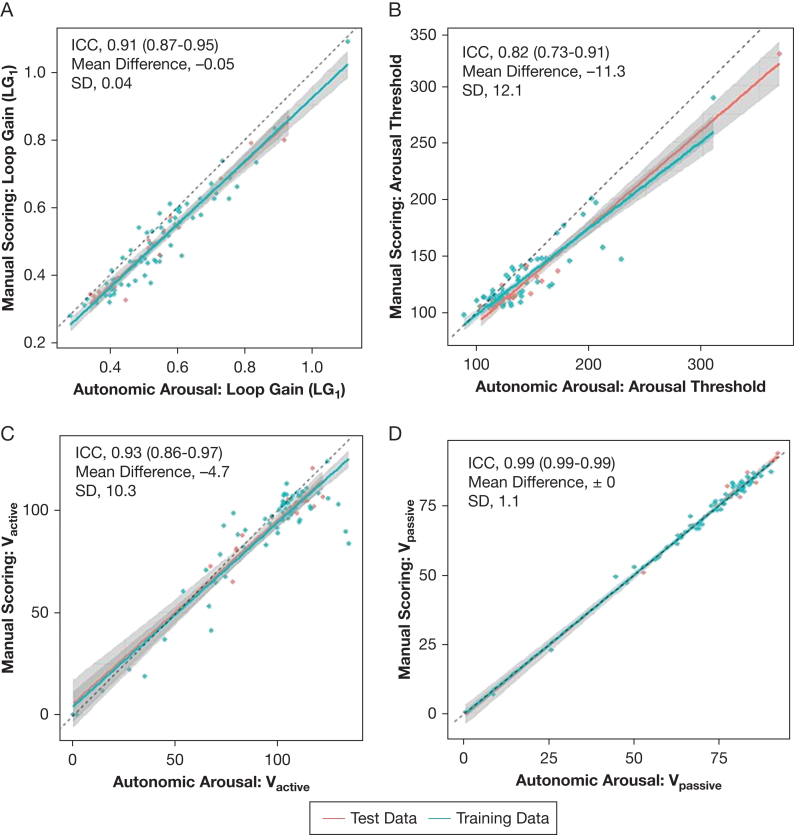
Scatterplots showing endotypic traits based on manually scored EEG arousals vs automatically derived autonomic arousals: LG_1_ (A), arousal threshold (B), V_active_ (C), and V_passive_ (D). The dashed line represents identity, and the solid line represents the linear fit with its 95% CI as a shaded area, separated by test (red; n = 17) and training (blue; n = 70) data. ICC, mean differences, and SD are reported for the entire cohort (n = 87). Although systematic differences were observed, high relative agreement was maintained. ICC = intraclass correlation coeffecient; LG_1_ = loop gain at 1 cycle/min; V_active_ = ventilation at active muscle activity; V_passive_ = ventilation at passive muscle activity.

Endotypic traits derived from the PUP_EEG_ reference model, using the manually scored cortical arousals, were compared with the PPG-based PUP_PPG_ model in a dedicated training set. A visual comparison of the method’s performance for key endotypic traits is presented in Figure 4. A Bland-Altmann plot and the distribution of pairwise differences in the test set are provided for loop gain at 1 cycle/min and arousal threshold (e-Fig 6). The method comparison is detailed in Table 4, including median and interquartile ranges for both outputs, their respective mean differences and SD, the results of a paired *t* test, and the ICC. The comparison revealed systematic mean differences in most endotypic traits except for delay and V_passive_. Reliability assessments using ICC showed high reliability (ICC > 0.80) for all traits, except ventilatory response to arousals. CIs for ICC were wider for arousal threshold, ventilatory response to arousals, and muscle compensation, requiring testing in larger test sets in future studies.

**Table 4:** Assessment of Agreement and Reliability Between the EEG-Derived and the PPG-Derived Endotyping Approach Based on the Designated Test Set (n = 17)

Variable	PUP_EEG_	PUP_PPG_	Difference	*P* Value	ICC (95% CI)
LG_1_	0.49 (0.36-0.57)	0.53 (0.42-0.60)	–0.05 (0.04)	< .001	0.95 (0.88-0.98)
LG_n_	0.43 (0.35-0.49)	0.44 (0.41-0.54)	–0.04 (0.03)	< .001	0.91 (0.84-0.95)
Arousal threshold	137 (132-152)	156 (141-170)	–15 (9)	< .001	0.85 (0.48-0.96)
VRA	39.5 (28.0-56.0)	18.5 (8.6-28.7)	20.5 (15.2)	< .001	0.51 (0.30-0.69)
Delay	14.4 (11.4-15.5)	13.9 (11.5-15.5)	0.1 (0.8)	.553	0.97 (0.93-0.99)
V_active_	101.5 (80.9-104.1)	104.6 (79.6-112.9)	–4.1 (7.1)	.029	0.96 (0.81-0.99)
V_passive_	95.0 (89.4-96.2)	95.1 (89.0-96.1)	0.0 (0.9)	.923	0.99 (0.99-0.99)
V_min_	61.3 (44.7-72.0)	63.6 (48.5-73.7)	–1.9 (1.8)	< .001	0.99 (0.99-0.99)
V_comp_	5.1 (0.0-7.9)	7.4 (0-19.2)	–4.1 (7.3)	.036	0.80 (0.60-0.91)

Data are presented as median (interquartile range) or mean (standard deviation) unless otherwise indicated. ICC = intraclass correlation coefficient; LG_n_ = loop gain at natural frequency; LG_1_ = loop gain at 1 disturbance/min; PPG = photoplethysmography; PUP = Phenotyping Using Polysomnography; PUP_EEG_ = established EEG-based PUP methodology; PUP_PPG_ = modified PPG-based PUP methodology; V_active_ = ventilation at active muscle activity; V_comp_ = compensatory ventilation; V_min_ = ventilation at minimum muscle activity; V_passive_ = ventilation at passive muscle activity; VRA = ventilatory response to arousals.

Sensitivity analysis showed expected effects for event characterization in non-rapid eye movement vs rapid eye movement sleep and supine vs non-supine body position in both the PUP_EEG_ and PUP_PPG_ models (e-Tables 5, 6). For example, loop gain was lower in rapid eye movement sleep than in non-rapid eye movement sleep, and upper airway collapsibility was higher in supine vs nonsupine positions. The arousal threshold aligned with previous descriptions of its relationship to other clinical metrics (e-Fig 7, e-Table 7).

## Discussion

Our study demonstrated that oximeter-derived pulse wave analysis of autonomic responses to respiratory events allows for the characterization of pathophysiologic endotypes in OSA with high accuracy in the absence of EEG-based arousal classification. This approach achieved strong reliability in key endotypic traits of chemosensitivity and upper airway collapsibility, suggesting that pulse wave analysis may provide clinically relevant insights into the pathophysiologic features of OSA without the need for EEG recordings. Our findings may impact the availability of automated endotyping for broader clinical applications substantially by using simple home sleep apnea tests (HSATs).

Our analysis provided a detailed overview of the autonomic pulse wave responses after respiratory disturbances in OSA. Previous research has explored the concept of analyzing autonomic responses to cortical arousals from sleep using various non-EEG signals, including peripheral arterial tonometry, to assess sympathetically mediated vasoconstrictions.^14^^,^^16^^,^^29^, ^30^, ^31^ Fluctuations in pulse wave amplitude and heart rate have been established since as surrogate markers for arousal-associated changes in sympathetic activity.^15^^,^^32^ In line with our findings, others reported reductions in finger blood flow (PWA) of 40% to 60% and increases in heart rate of 10% to 20% in response to arousals, which are more pronounced in apneas compared with hypopneas.^25^^,^^33^ Other previously identified hemodynamic responses to arousal included reductions in pulse transit time and increases in BP,^14^^,^^34^^,^^35^ which may be reflected by an altered PPT.^36^ Indeed, our results show for the first time an association between the presence of arousals and spontaneous changes in PPT. This is particularly relevant because PPT may capture subtle changes in vascular tone that are not readily apparent in PR or PWA alone. Additionally, previous work emphasized the effect of the type of event and desaturation on the variation in pulse wave responses.^25^ Specifically, prior work has linked event-related hypoxic burden and vasoconstriction drops, VCD_Ev_, a metric analogous to the PWA reduction analyzed in this study.^24^ By incorporating multiple parameters with an independent significant relationship to the presence of arousals, our model may offer a more comprehensive assessment of autonomic arousals. Besides the cardiovascular responses to arousals, it is well documented that arousals from sleep have various effects on respiratory metrics, such as respiratory rate and ventilatory overshoot, which could be used to predict the presence of arousals.^37^^,^^38^ We performed additional supplementary analyses confirming a linear relationship between the magnitude of autonomic and ventilatory responses (e-Table 8). However, we excluded variables describing ventilatory magnitude from our prediction model because the primary aim of the subsequent endotyping is to differentiate surges in ventilation driven by chemical drive vs arousal-related wakefulness drive. In summary, our approach simultaneously integrates various components of the autonomic response—including changes in PWA, PR, and PPT, as well as the related SpO_2_ reduction and the type of events—to establish the usefulness of oximeter-derived metrics for an endotypic characterization. Future iterations could benefit from incorporating other, more nuanced respiratory features, such as the morphologic features of individual flow-limited breaths.^39^^,^^40^

Despite the recognition of autonomic responses to arousals, their use in clinical practice has remained limited. Although the classification of autonomic arousals has been used to identify hypopneas in HSAT, the performance in classifying arousals remained moderate.^17^^,^^18^ The present study builds on this context, combining recent advances in pulse wave analysis and respiratory physiology to integrate the concept of autonomic arousals into the PUP model for estimating endotypic traits of OSA. Although the already documented limitations of arousal detection by autonomic signals also persist in our analysis, as indicated by comparably low sensitivity and specificity for the arousal prediction alone, several important aspects create significant robustness in our approach. First, the evaluation for endotypic traits is based on a respiratory model during airway obstructions. Although various physiologic conditions, such as sleep stage transitions or nonrespiratory sleep disorders, may create variability in autonomic activity, our analysis solely addresses the responses to a respiratory event. Thus, other factors, such as spontaneous arousals or leg movements, did not influence the performance of our model. Second, the PUP model does not rely solely on individual events, but rather evaluates respiratory patterns within a 7-minute time frame. Thus, the presence or absence of a single arousal had only a minor influence on the overall classification of the algorithm. Finally, our data confirmed that the magnitude of autonomic responses is related to the ventilatory response (e-Table 8). Thus, the likelihood of autonomic arousals is higher for events with a high ventilatory overshoot. By capturing such events more accurately, the overall results likely will be more robust because of the mathematical modeling of ventilatory responses as the sum of chemical and wakefulness drive.^9^ Our presented results support this reasoning: although the predictive capability of the presented approach to label arousals compared with manual scoring showed moderate sensitivity and specificity of 0.71 and 0.59, respectively, most endotypic characteristics showed high reliability in terms of ICC. Less reliability and wider CIs were observed for arousal threshold and ventilatory response to arousals. These important features require additional investigations to conclude their usefulness in the provided PPG-based approach. In additional supplementary sensitivity analyses, the derived endotypes further confirmed the capability to distinguish certain physiologic features based on sleep stage and position (e-Tables 5, 6). They aligned with previous descriptions of pathophysiologic characterizations (e-Fig 7, e-Table 7). It is important to note that systematic differences between endotypes derived from manual and automatic arousal classification were observed. For example, loop gain derived from autonomic arousals generally is larger than the polysomnography-derived reference. However, the high ICC suggests a strong relative agreement, indicating that both methods can differentiate high and low values on this scale reliably. Future validations performed in clinical cohorts need to evaluate the clinical relevance of these differences.

### Strengths and Limitations

Several important strengths of the study need to be mentioned. To our knowledge, this is the first description of an approach using advanced pulse wave analysis to characterize pathophysiologic endotypes in OSA. We showed excellent reliability in key endotypic characteristics of OSA, such as loop gain and collapsibility (V_min_, V_passive_). Good reliability also was found for the arousal threshold and muscle compensation. The study used clinically collected data and state-of-the-art polysomnography, including manual scoring by trained experts. The final results of our approach were derived by using only the respiratory flow signal and the PPG-derived pulse waveform and oxygen saturation. Using only data from 2 sensors in the analysis has a specific value because these typically are available for various HSAT devices. This further expands the availability of endotypic characterizations with acceptable precision.

It is important to acknowledge certain limitations of this study. First, the algorithm relied on an arousal prediction derived from the same cohort, which requires additional validation studies. These studies should include larger validation sets to assess the generalizability of the model and incorporate gold standard references, such as ventilatory drive assessed by diaphragm electromyography or therapeutic interventions. Second, the sensitivity and specificity of autonomic arousal prediction were 0.71 and 0.59, respectively. Despite this moderate agreement, the PUP_PPG_ showed a strong and robust agreement in derived endotypic characteristics with the reference PUP_EEG_ model. Finally, the current approach for PUP_PPG_ evaluation did not include patient-specific information (eg, comorbidities or medication) that may influence autonomic activity, and therefore may cause additional between-patient variability. This potential variability is addressed partly by using a patient-specific search window, which controls for differences in circulatory time delays.^41^ In addition, the use of relative changes, expressed as % change in relation to pre-event baseline, controls for patient-specific baseline differences. Hence, this study serves as an initial methodologic and conceptual foundation to explore further the usefulness of incorporating PPG-based pulse wave features in the pathophysiologic endotyping of OSA.

## Interpretation

This study demonstrated the potential of oximeter-derived pulse wave analysis to characterize pathophysiologic endotypes in OSA without an overnight EEG recording. The ability to derive endotypic traits from readily available pulse wave signals offers a promising path for expanding the accessibility and applicability of OSA endotyping. This approach could enable the integration of automated endotyping into a wide range of clinical settings, particularly the screening of OSA using HSAT devices. This work contributes to the advancement of personalized precision medicine for the treatment and management of OSA.

## Funding/Support

The study was supported by Desitin GmbH, Hamburg, Germany (CAISA study program); the 10.13039/501100003793Swedish Heart and Lung Foundation [Grants 20180585, 20210529, and 20210500]; and the Swedish state under the agreement between the Swedish government and the county councils, the ALF-agreement [Grants LUA/ALF 966319 and 966283]. C. S. is a PhD student financed by the University of Gothenburg, Sahlgrenska Academy [Grant PAR 2020/1175]. S. A. S. was funded by the 10.13039/100000050National Heart, Lung, and Blood Institute, 10.13039/100000002National Institutes of Health [Grants R01HL168067 and R01HL146697]. The contributions of Jan Hedner, Ding Zou, and Ludger Grote was supported by an EU Horizon 2020 grant [Grant “Sleep Revolution” 965417].

## Financial/Nonfinancial Disclosures

The authors have reported to *CHEST* the following: J. H. reports lecturing activities for SomnoMed, Desitin, and Bayer Pharma; is also a collaborator in the European Union Horizon 2020 and EUROSTAR programs (Sleep Revolution, ‘WatchIT); has received grants from European Union interreg program Sleep Aross Waters; is an advisory committee member for SomnoMed; and is a co-owner of a licensed patent for sleep apnea treatment. S. A. S. served as a consultant to Nox Medical, Inspire, Merck, Apnimed, Eli Lilly, and Respicardia and has received grant support from Apnimed, Prosomnus, and Dynaflex; his industry interactions are managed by the Brigham and Women’s Hospital. Through his institution, he is also named coinventor on intellectual property pertaining to combination pharmacotherapy (including royalties) and OSA phenotyping using wearable technology. The PUP software and methods are freely available for academic use. L. G. reports lecturing activities for Resmed, Philips, Astra Zeneca, and Lundbeck as well as grant support for scientific projects from Bayer and the European Union Horizon 2020 and EUROSTAR programs (Sleep Revolution, APNEWAY); is an advisory committee member for Onera; and is a co-owner of a licensed patent for sleep apnea treatment. None declared (C. S., D. Z.).

## Supplementary Data

e-Online Data
