# Supplementary material for: From Pulse to Phenotype: Sleep Apnea Endotyping for Polygraphy via Oximeter-Derived Autonomic Arousal
Source: Chest. 2025 Dec 18;169(5):1345–56. doi: 10.1016/j.chest.2025.12.005 (PMC13197968; doi:10.1016/j.chest.2025.12.005)
Supplement: e-Online Data [file mmc1.docx]

**From Pulse to Phenotype: Oximeter-derived autonomic arousal detection for sleep apnea endotyping**

**Online Supplement**

Christian Strassberger^1^, Jan Hedner^1,2^, Scott A. Sands^3^, Ding Zou^1^, Ludger Grote^1,2^

Affiliations:

^1^Center for Sleep and Vigilance Disorders, Institute of Medicine, Sahlgrenska Academy, University of Gothenburg, Gothenburg, Sweden

^2^Center for Sleep Medicine, Department of Respiratory Medicine, Sahlgrenska University Hospital, Gothenburg, Sweden

^3^Division of Sleep and Circadian Disorders, Brigham and Women's Hospital and Harvard Medical School, Boston, MA.

**Address for correspondence**

Christian Strassberger, M.Sc.

Center for Sleep and Vigilance Disorders

Sahlgrenska Academy, University of Gothenburg

Medicinaregatan 8B, Box 421

40530 Gothenburg

Sweden

Email: [christian.strassberger@gu.se](mailto:christian.strassberger@gu.se)

**Table of Content**

1. **Algorithmic Realization**
   1. **Derivation of Pulse Wave Features**
   2. **Derivation of Autonomic Responses**
2. **Sensitivity Analyses**
   1. **Independent Association of Pulse Wave Features**
   2. **Analysis of Multicollinearity**
   3. **Selection of Prediction Cutoff**
   4. **Variation by Sleep Stages and Body Position**
   5. **Arousal Threshold based on clinical predictors**
   6. **Respiratory Responses**
3. **Algorithmic Realization**

This section gives a detailed description of the implementation of the pulse wave analysis used in the present work. This includes,

1. Details on pulse wave extraction, decomposition, parameter derivation, plausibility checks, and artifact detection using the pattern-matching approach
2. Derivation of Autonomic Responses using individual search windows
3. **Derivation of Pulse Wave Features**

Initially, the input signal is split into chunks of 10-minute length, which are processed in parallel. In each chunk, a low-pass filter with a 2 Hz cutoff is applied to identify the position of individual pulse waves. For each of these candidates, a pulse wave decomposition was performed utilizing a model-based curve-fitting approach, as suggested in other applications before^1^. For this purpose, two Gaussian curves are determined, whose sum represents the recorded pulse wave (**e-Figure 1**). The Gaussian curves represent the heart ejection as well as the reflection wave. The center of the first wave is used to identify the pulse wave amplitude (PWA) of the main pulse beat, while the distance between the two centers represents the pulse propagation time (PPT). Pulse rate (PR) is derived from the nadir (start/end) between detected pulse waves. Additionally, the squared difference between the best model fit and the recorded signal is derived and used for artifact detection. As exemplified in the right panel of **e-Figure 1**, this method is specifically robust in detecting pulse wave features, even in the presence of noisy signals, which commonly occur in clinical routine assessments of the PPG pulse wave. Finally, a set of plausibility checks is performed, addressing the shape and magnitude of individual pulse wave features derived from the decomposition analysis. These plausibility checks, together with the residual of the pulse wave decomposition, are subsequently used for artifact detection.


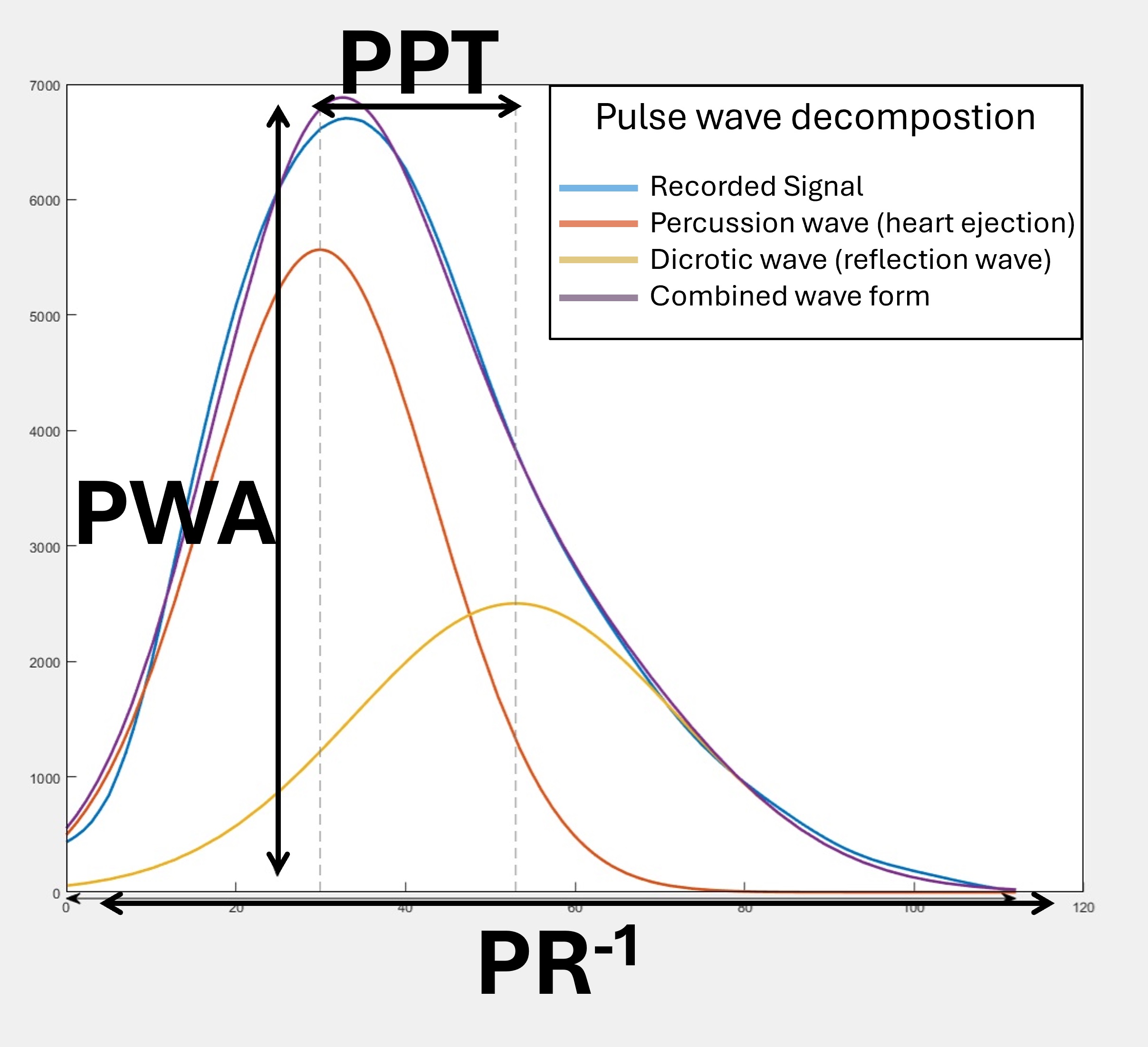

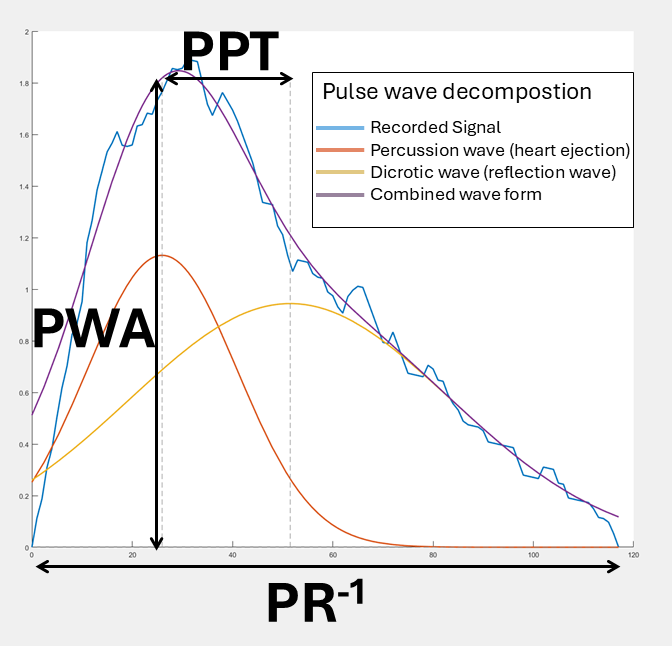


e-Figure 1. Visualization of the derived parameters from each pulse wave, utilizing a curve fitting approach: Two Gaussian curves (red, yellow) are determined, so that their sum (purple) approximates the shape of the recorded signal (blue). The centers of the two Gaussians are used to determine PWA and PPT. Pulse rate is based on the length between the present and the next pulse beat. The right panel emphasizes the capability of the algorithm to find appropriate estimations, even for low-quality pulse wave recordings.

Following the evaluation of each signal chunk, continuous signal traces are created for PWA, PPT, and PR and used for subsequent analyses. Artifacts, spanning over five or fewer consecutive pulse beats, were linearly interpolated. The continuous signals were up-sampled to a common signal frequency and smoothed using a Butterworth filter to remove high-frequency noise. A visualization of the process for PWA is presented in **e-Figure 2** below, showing the individual beat-to-beat pulse wave amplitude in green, together with the up-sampled and smoothed PWA-signal trace in red.


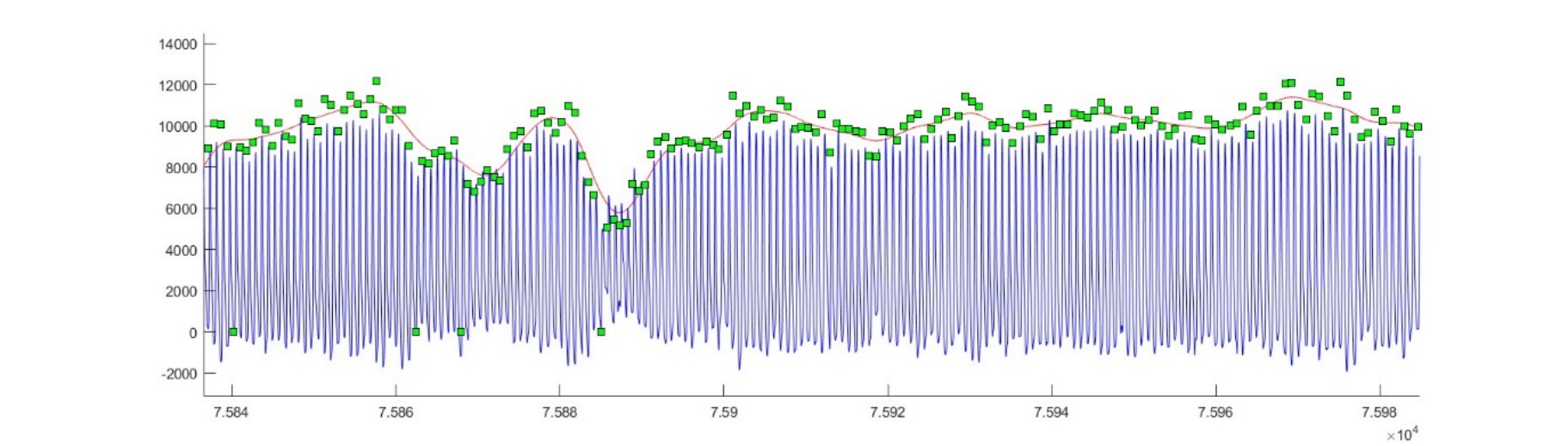


e-Figure 2. PWA: Beat-to-beat values (green), and the smoothed PWA signal trace (red), visualized together with the raw PPG signal (blue). Interpolated artifacts, due to difficulties in recognizing the pulse wave shape, are visualized with an amplitude value of 0 and treated as missing values.

1. **Derivation of Autonomic Responses**

Responses to respiratory events in PPG-derived signals may be influenced by a variety of factors, such as circulatory delay, individual intensity of arousals, and the overall responsiveness of the autonomic nervous system. Therefore, it is difficult to generalize the exact time frame in which an autonomic response to the respiratory event is expected. For this purpose, we implemented an approach that has been proposed in similar contexts before^2,3^: First, all respiratory events are extracted, together with the PPG-derived signals, and overlaid by centering the signals at the end of the respiratory event. In the resulting ensemble average, the individuals' characteristic response time is extracted based on the local minima surrounding the respiratory event. This patient-specific response window is then applied to all respiratory events to derive the respective autonomic responses. The approach is visualized in **e-Figure 3.**


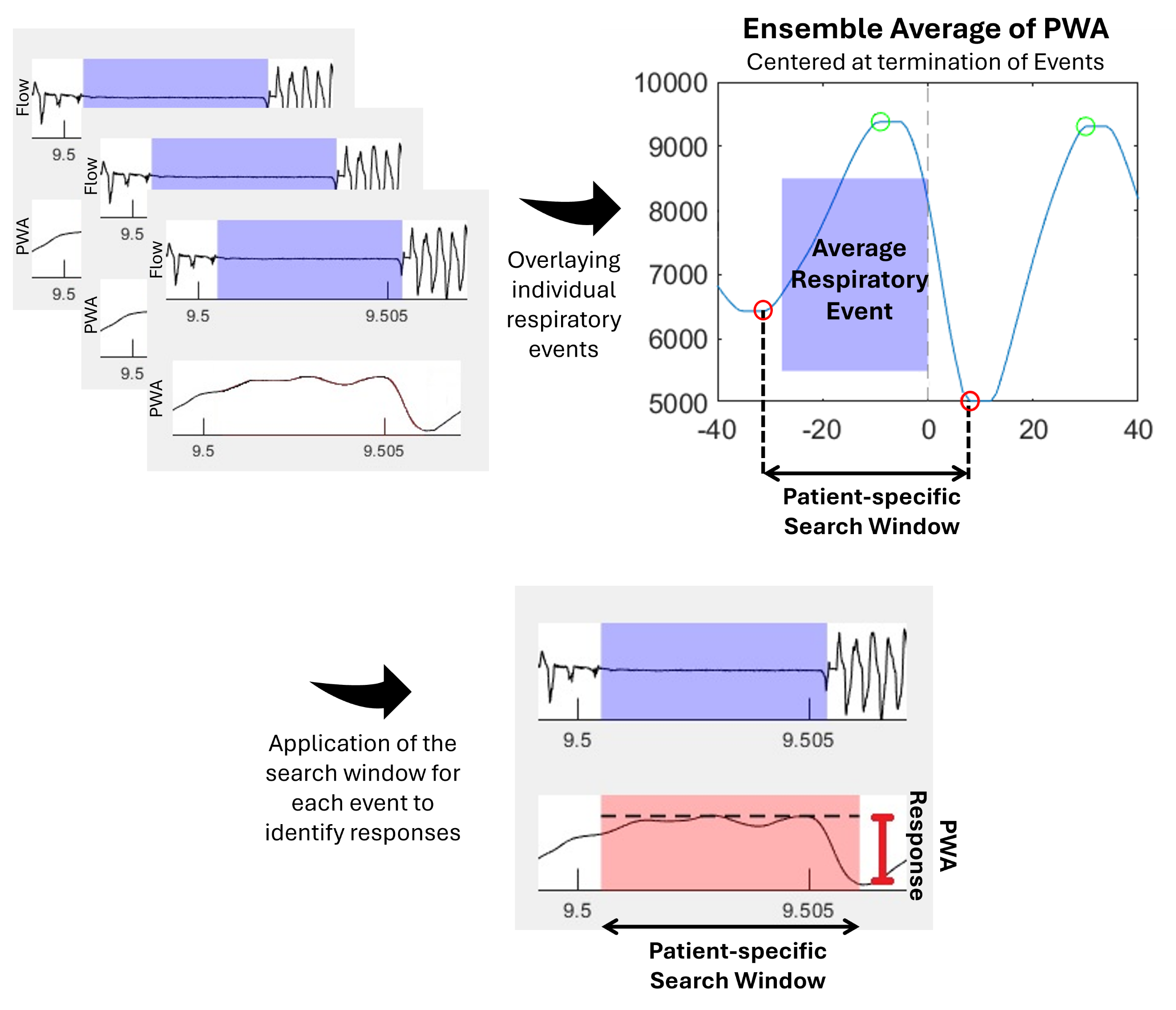


e-Figure 3. Visualization of the search window approach to determine individual responses. For each patient, all respiratory events are overlayed, centered at the end of the respiratory event (ensemble average). The search window is determined based on the local minima surrounding the respiratory event. The reduction in PWA is then determined within the patient-specific search window

1. **Sensitivity Analyses**

We conducted a series of sensitivity analyses to further validate the robustness and generalizability of our primary findings. These analyses aimed to further confirm the validity of our modeling approach by demonstrating consistency with previous research. Specifically, we explored the potential influence of sleep stage and body position on the model outcome and confirmed the relationship between the intensity of autonomic responses and breathing. Additionally, we compared the arousal threshold derived from our model with a different approach^4^, which utilizes clinical predictors to estimate a low arousal threshold.

**a. Independent Association of Pulse Wave Features**

To identify predictors of the presence or absence of arousals, a mixed-effects logistic regression analysis was employed, with subjects treated as random effects. Initially, a comprehensive model was constructed, including potential confounding factors (age, gender, body mass index (BMI)), alongside the primary pulse wave-derived predictors (changes in PR, PWA, PPT), the associated SpO2 desaturation, and type of event (apnea or hypopnea) **(e-Table 1)**. This model aimed to establish the independent association of the pulse wave parameters with the outcome.

|  | **Estimate** | **Std. Error** | **z value** | **p-value** |
| --- | --- | --- | --- | --- |
| **(Intercept)** | -1.300 | 0.864 | -1.504 | 0.132 |
| **Age** | 0.024 | 0.008 | 3.062 | **0.002** |
| **Gender Female** | 0.068 | 0.220 | 0.310 | 0.757 |
| **BMI** | -0.044 | 0.027 | -1.668 | 0.095 |
| **Event Hypopnea** | 0.151 | 0.051 | 2.944 | **0.003** |
| **SpO2 Change (%SpO2)** | 0.060 | 0.009 | 6.490 | **<0.001** |
| **PWA change (%)** | -0.034 | 0.001 | -25.238 | **<0.001** |
| **PR change (%)** | 0.043 | 0.003 | 14.789 | **<0.001** |
| **PPT change (%)** | -0.012 | 0.002 | -5.378 | **<0.001** |

e-Table 1 – *Linear mixed effect model to predict the presence of manually EEG-scored, cortical arousals. Subjects are used as random effect, while the presented parameters represent the fixed effects. Abbreviations: BMI – body mass index, PWA – pulse wave amplitude, PR – pulse rate, PPT – pulse propagation time;*

**b. Analysis of Multicollinearity**

To perform an evaluation of collinearity between the predictors used in the model deriving autonomic arousals, we evaluated the pairwise Pearson’s correlation coefficient (**e-Table 2**) as well as the Variance Inflation Factor (VIF) (**e-Table 3**).

Overall, the intercorrelation was low to moderate, with no indication of a bias affecting the prediction model.

|  | **SpO2 Change (%SpO2)** | **PWA Change (%)** | **PR Change (%)** | **PPT Change (%)** |
| --- | --- | --- | --- | --- |
| **SpO2 Change (%SpO2)** | - | -0.253 | 0.287 | -0.171 |
| **PWA Change (%)** | -0.253 | - | -0.356 | 0.091 |
| **PR Change (%)** | 0.287 | -0.356 | - | -0.238 |
| **PPT Change (%)** | -0.171 | 0.091 | -0.238 | - |

e-Table 2. Pearson’s correlation coefficients between PPG-derived predictors for the presence of arousals.

|  | **Variance Inflation Factor** |
| --- | --- |
| **Age** | 1.03 |
| **Gender** | 1.04 |
| **BMI** | 1.01 |
| **Event Hypopnea** | 1.15 |
| **SpO2 Change** | 1.19 |
| **PWA Change** | 1.10 |
| **PR Change** | 1.13 |
| **PPT Change** | 1.03 |

e-Table 3. Variance Inflation Factors for the model predicting the presence of arousals, controlling for confounders

**c. Selection of Prediction Cutoff**

During internal validations of the approach, we noticed that the utilization of the Youden Index resulted in a less precise classification of endotypic traits than a more sensitive, less specific threshold. We attribute this outcome to the fact that with the higher threshold corresponding to the Youden Index, the prevalence of predicted arousal events (57%) is much lower than in the manual scoring (79%). Missing an arousal leads to underestimating the total ventilatory drive, as the model would falsely attribute the entirety of the subsequent hyperventilatory response solely to chemical stimuli. This underestimation can significantly mischaracterize key endotypic traits that are sensitive to the magnitude and dynamics of ventilatory responses, such as loop gain (LG), ventilatory response to arousals (VRA), and arousal threshold (ArTh). Conversely, while a false positive prediction might lead to a slight overestimation of the wakefulness-related drive component, this appears to be less disruptive to the overall characterization of the patient’s ventilatory control system. Overall, false negative arousal predictions appear to have more substantial consequences on the derived endotypic traits than false positive predictions.

To determine the final prediction cutoff, a weighted Youden Index was calculated (**e-Figures 4-5)**, weighting sensitivity with a factor of 1.25 compared to specificity, i.e.,

$$\max\left( \frac{5}{9}\times Sensitivity+\frac{4}{9}\times Specificity \right)$$

**E-tables 4a-b** contrast the true/false predictions of the model using the unbalanced Youden Index, as well as the weighted Youden Index.


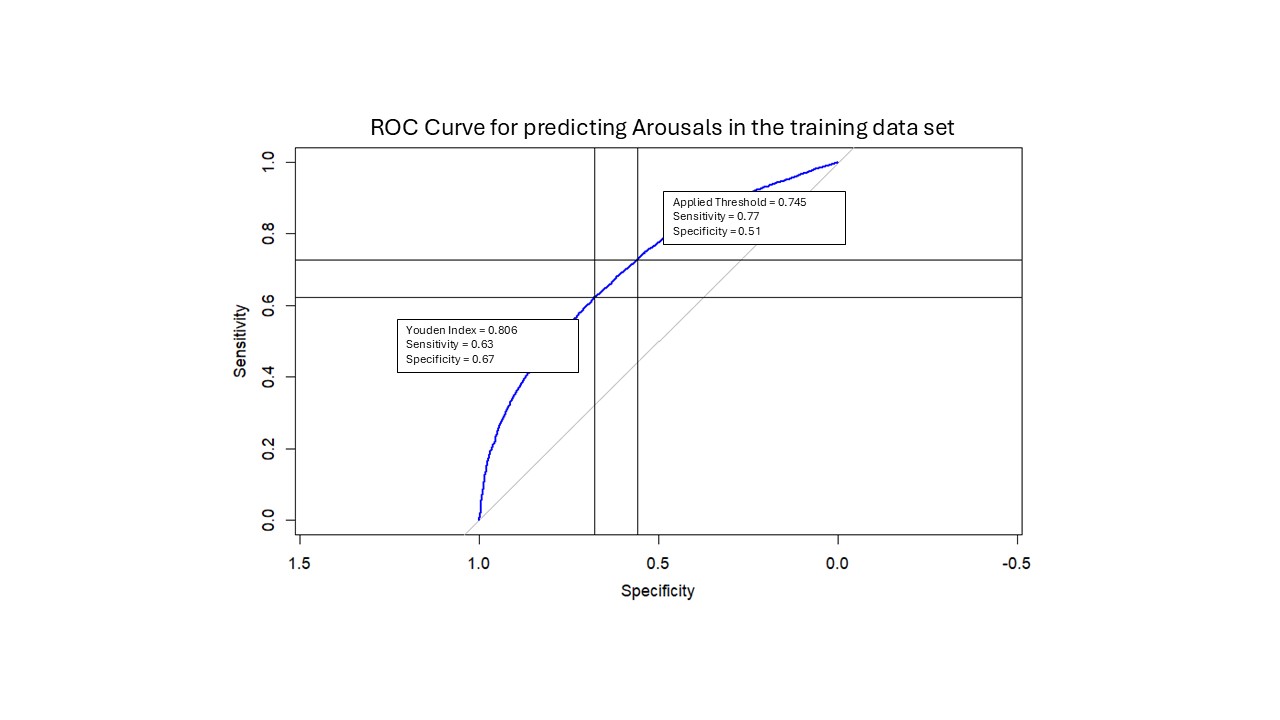


e-Figure 4. Visualization of the weighted prediction threshold of 0.745 and the Youden index of 0.806, derived from the training dataset. Using the prediction cutoff of 0.745, the sensitivity and specificity of the arousal prediction were 0.77 and 0.51. The Youden Index is found at 0.806 with sensitivity and specificity of 0.63 and 0.67, respectively.


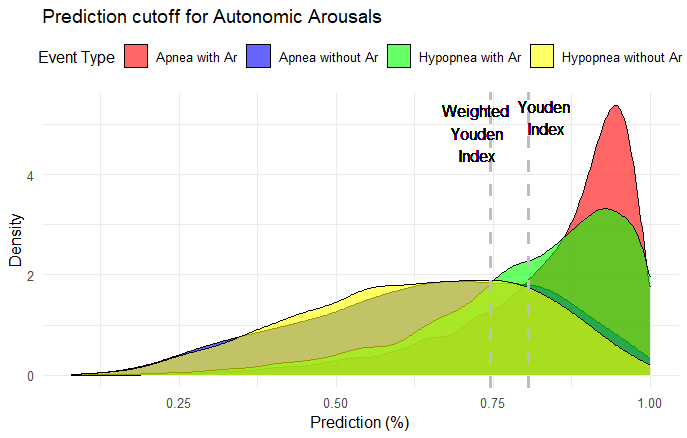


e-Figure 5 - *Overview of the predicted probability for arousals, separated by event type and presence of arousals. The prediction was based on coefficients derived from a linear mixed-effects model, including autonomic and hypoxic event responses and the event type. A weighted Youden Index was used to predict the presence of arousals in the final model, which was found at 0.745. For reference, the unweighted Youden Index at 0.806 is visualized. Manually classified events are depicted by different colors. Events with a predicted probability beyond the prediction threshold are classified as events with autonomic arousals, resulting in a sensitivity of 0.71 and a specificity of 0.51. Abbreviations: Ar - Arousal*

| **Prediction cutoff: 0.745** | **Autonomic: No Arousal** | **Autonomic: Arousal** |
| --- | --- | --- |
| **Manual: No Arousal** | 1611 | 1522 |
| **Manual: Arousal** | 2633 | 8734 |

e-Table 4a. True/false predictions of the presence of arousals following apnea/hypopnea events in the training data, using the weighted prediction cutoff of 0.745. Using this threshold, 71% of events are classified as terminated by arousal.

| **Prediction cutoff: 0.806** | **Autonomic: No Arousal** | **Autonomic: Arousal** |
| --- | --- | --- |
| **Manual: No Arousal** | 2096 | 1037 |
| **Manual: Arousal** | 4149 | 7218 |

e-Table 4b. True/false predictions of the presence of arousals following apnea/hypopnea events in the training data, using the Youden index as prediction cutoff at 0.806. Using this threshold, 57% of events are classified as terminated by arousal.


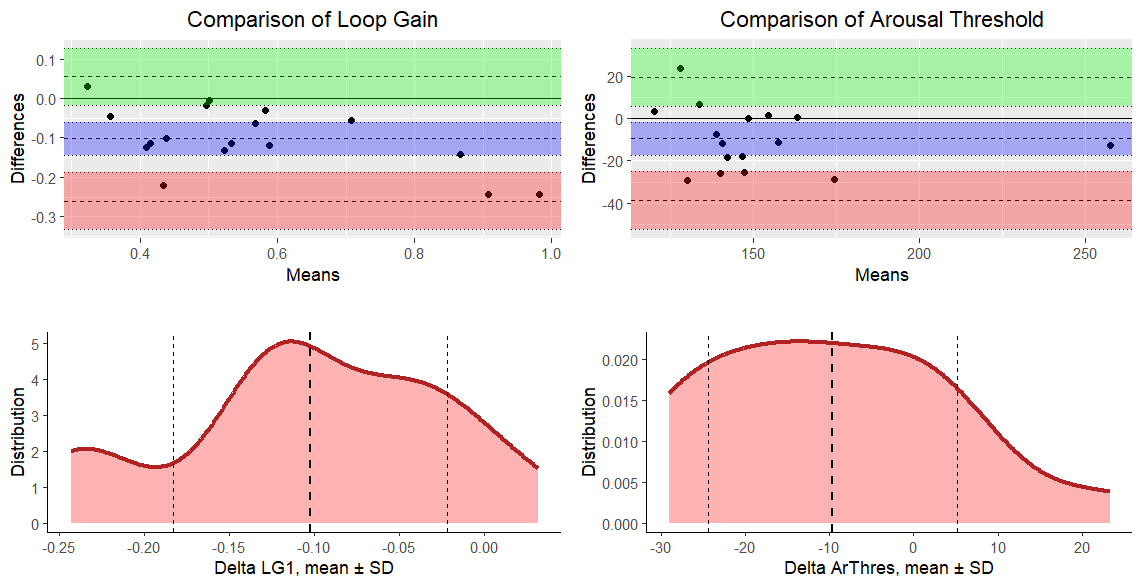
e-Figure 6 - *Bland-Altmann plots (top row) and the distribution of differences (bottom row) for loop gain and arousal threshold, utilizing the data from the independent test dataset (n = 17). The data shown addresses the difference between endotypic traits derived using the PSG-based reference algorithm and the new approach utilizing autonomic arousals. Abbreviations: LG_1_ – loop gain, ArThres – Arousal Threshold, SD – standard deviation;*

**d. Variation by Sleep Stages and Body Position**

Based on the previous evaluations of sleep physiology, it is assumed that measures of loop gain and collapsibility vary based on sleep stages and position. This includes a lower loop gain during REM sleep, a higher loop gain during supine position, and higher collapsibility (lower Vpassive) in supine position^5,6^. We restricted the analysis of the PSG-derived, as well as the PPG-derived endotypic traits by sleep stage and position to evaluate whether the expected differences can be replicated in our data using the new approach. An overview of the analysis is presented in **e-Tables 5-6**. Median and IQR are presented by sleep stage and position, together with a paired t-test assessing their difference. In summary, endotypes based on the PUPBeta_PPG_ showed the expected changes during REM sleep and supine position.

|  | REM: Median | IQR | NREM:  Median | IQR | p-value |
| --- | --- | --- | --- | --- | --- |
| LG1 | 0.43 | 0.38 - 0.51 | 0.59 | 0.49 – 0.65 | < 0.001 |
| LGn | 0.45 | 0.40 - 0.52 | 0.48 | 0.43 – 0.54 | 0.083 |
| delay | 16.4 | 14.5 - 19.4 | 12.9 | 11.4 – 15.1 | < 0.001 |
| VRA | 18.8 | 6.9 - 34.3 | 22.4 | 12.5 – 31.9 | 0.312 |
| ArThres | 153 | 134 - 178 | 155 | 142 - 168 | 0.959 |
| Vpassive | 91.4 | 73.9 - 96.3 | 94.1 | 89.4 - 97.1 | < 0.001 |
| Vactive | 99.8 | 67.3 - 104.2 | 103.3 | 89.0 – 110.1 | 0.023 |
| Vcomp | 5.9 | 0 - 14.5 | 7.9 | 0 – 15.5 | 0.435 |
| Vmin | 63.4 | 37.6 - 72.5 | 63.7 | 44.6 – 74.8 | 0.695 |

e-Table 5. Overview of different physiological traits separated by REM and NREM sleep, utilizing data from the entire cohort (n=87). All parameters are derived using the PPG-derived arousal prediction. As hypothesized, loop gain values are lower in REM sleep, indicating more stable ventilatory control.

**e. Arousal Threshold based on clinical predictors**

The potential to quantify pathophysiological traits of OSA has been investigated before by Edwards et al^4^. In this study, a precise measure of respiratory effort was performed using an epiglottic pressure catheter to derive a measure of arousal threshold. The work identified predictors from clinical routine PSG and derived a simple criterion to predict low arousal threshold. Specifically, low arousal threshold was predicted by fulfilling 2 out of 3 criteria, i.e.,

- AHI < 30 events / h

|  | Supine  Median | IQR | Non-Supine  Median | IQR | p-value |
| --- | --- | --- | --- | --- | --- |
| LG1 | 0.57 | 0.47 – 0.73 | 0.50 | 0.42 – 0.58 | < 0.001 |
| LGn | 0.48 | 0.41 – 0.55 | 0.45 | 0.41 – 0.51 | 0.080 |
| delay | 13.3 | 10.9 – 15.4 | 14.9 | 12.2 – 17.4 | < 0.001 |
| VRA | 26.2 | 16.3 – 47.5 | 18.2 | 8.1 – 27.7 | < 0.001 |
| ArThres | 157 | 142 - 181 | 147 | 134 - 160 | < 0.001 |
| Vpassive | 87.9 | 71.1 – 94.5 | 95.5 | 91.3 – 98.1 | < 0.001 |
| Vactive | 98.2 | 57.0 – 104.5 | 104.4 | 99.3 – 111.4 | < 0.001 |
| Vcomp | 6.2 | -1.2 – 13.1 | 9.6 | 3.9 – 16.6 | 0.132 |
| Vmin | 53.8 | 20.4 – 69.1 | 69.5 | 54.9 – 75.4 | < 0.001 |

e-Table 6. Overview of different physiological traits separated by supine and non-supine position, utilizing data from the entire cohort (n=87). All parameters are derived using the PPG-derived arousal prediction. As hypothesized, measures of collapsibility (i.e., lower Vpassive) indicate a higher upper airway collapsibility during the supine position.

- Fraction of hypopnea > 58.3 %
- Average nadir in SpO2 > 82.5 % associated with respiratory events

We applied these criteria to identify subjects classified as low arousal threshold, and compared their PUP-derived arousal threshold, applying both the established PUPPSG and the pulse wave-augmented PUP_PPG_ method. A strong overlap between PSG-based low arousal threshold classification, and lower numerical values in PUP-derived arousal threshold was found (Mean PUP_PSG_ for high/low = 155 and 113, respectively; Mean PUP_PPG_ for high/low = 172 and 123, respectively). An arbitrary cutoff of 140% eupneic ventilation was used for visualization purposes. The results from a direct comparison between the two methodologies are shown in **e-Figure 7** and **e-Table 7**.

**
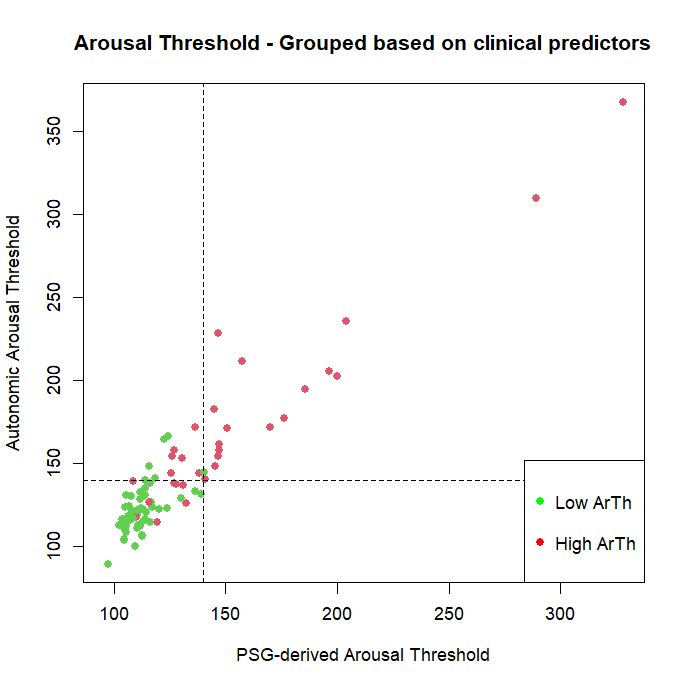
**

e-Figure 7. Visualizion of PSG and PPG-derived arousal threshold on the x- and y-axis, together with the high and low arousal threshold classification based on Edwards et al. in green and red colour. The dashed line represents the selected reference value at 140% eupneic ventilation.

| **Autonomic Arousal** | | |  | **Cortical Arousal** | | |
| --- | --- | --- | --- | --- | --- | --- |
|  | **High ArTh** | **Low ArTh** |  |  | **High ArTh** | **Low ArTh** |
| **ArTh_PPG_ > 140% eupnea** | 23 | 5 |  | **ArTh_PSG_ > 140% eupnea** | 17 | 1 |
| **ArTh_PPG_ <= 140% eupnea** | 9 | 48 |  | **ArTh_PSG_ <= 140% eupnea** | 15 | 54 |

e-Table 7. Number of subjects, classified as high or low arousal threshold according to Edwards et al., stratified by PPG and PSG-derived arousal threshold, applying a threshold of 140% of eupneic ventilation.

**f. Respiratory Responses**

According to the hypothesis, the ventilatory response to apneic or hypopneic respiratory events is assumed to be related to pulse wave-derived metrics predicting the presence of arousals. To test this relation, we defined parameters V_before_ and V_after_ as the mean volume of 3 breaths before or after a respiratory event based on a breath-by-breath analysis of the respiratory pressure signal using a nasal cannula. We further defined the normalized ventilatory response as V_diff_ := (V_after_ – V_before_) / V_eupnea_, where V_eupnea_ is the mean respiratory volume in a 7-minute moving average window. Response in PWA was defined as the percentage change following respiratory events as described in the main text.

PWA response and V_diff_ were significantly correlated, with a correlation coefficient r = -0.3, p < 0.001. We further employed a linear model (**e-Table 8**), with V_diff_ as the dependent variable, and the PPG responses in PWA, PPT, and PR as predictors, together with the type of respiratory event (apnea / hypopnea). All predictors confirmed an independent, significant relation with the outcome, confirming their use in the respiratory analysis of the PUP model.

Dependent: V_diff_

Coefficients:

Estimate Std. Error t value Pr(>|t|)

(Intercept) 0.3968448 0.0350407 11.325 < 2e-16 ***

PWA Delta (%) -0.0151117 0.0005675 -26.628 < 2e-16 ***

PPT Delta (%) 0.0030096 0.0008719 3.452 0.000558 ***

PR Delta (%) 0.0214200 0.0008166 26.230 < 2e-16 ***

Event Hypopnea -0.6014991 0.0196250 -30.650 < 2e-16 ***

e-Table 8. Overview of the linear model to predict ventilatory response to respiratory events, using pulse wave-derived features and the type of event.

**References**

1. Nagasawa T, Iuchi K, Takahashi R, et al. Blood pressure estimation by photoplethysmogram decomposition into hyperbolic secant waves. *Applied Sciences.* 2022;12(4):1798.

2. Azarbarzin A, Sands SA, Younes M, et al. The Sleep Apnea-Specific Pulse-Rate Response Predicts Cardiovascular Morbidity and Mortality. *Am J Respir Crit Care Med.* 2021;203(12):1546-1555.

3. Azarbarzin A, Sands SA, Stone KL, et al. The hypoxic burden of sleep apnoea predicts cardiovascular disease-related mortality: the Osteoporotic Fractures in Men Study and the Sleep Heart Health Study. *Eur Heart J.* 2019;40(14):1149-1157.

4. Edwards BA, Eckert DJ, McSharry DG, et al. Clinical predictors of the respiratory arousal threshold in patients with obstructive sleep apnea. *Am J Respir Crit Care Med.* 2014;190(11):1293-1300.

5. Joosten SA, Landry SA, Sands SA, et al. Dynamic loop gain increases upon adopting the supine body position during sleep in patients with obstructive sleep apnoea. *Respirology.* 2017;22(8):1662-1669.

6. Landry SA, Andara C, Terrill PI, et al. Ventilatory control sensitivity in patients with obstructive sleep apnea is sleep stage dependent. *Sleep.* 2018;41(5).
